# Annotating the pangenome reveals the diversity in the genetic basis for metabolic enzymes

**DOI:** 10.1126/sciadv.aeb3363

**Published:** 2026-07-01

**Authors:** Omid Ardalani, Patrick V. Phaneuf, Kalpathy J. Krishnan, David Pride, Lars K. Nielsen, Bernhard O. Palsson

**Affiliations:** ^1^ Novo Nordisk Foundation Center for Biosustainability, Technical University of Denmark, Lyngby, Denmark.; ^2^ Department of Bioengineering, University of California, San Diego, La Jolla, CA, USA.; ^3^ Department of Pathology, University of California, San Diego, La Jolla, CA, USA.; ^4^ Australian Institute for Bioengineering and Nanotechnology, The University of Queensland, Brisbane, Queensland, Australia.; ^5^ Bioinformatics and Systems Biology Program, University of California, San Diego, La Jolla, CA, USA.; ^6^ Department of Pediatrics, University of California, San Diego, La Jolla, CA, USA.; ^7^ Center for Microbiome Innovation, University of California, San Diego, La Jolla, CA, USA.

## Abstract

Affordable sequencing has flooded public databases with bacterial genomes; yet, species-scale maps that connect gene content variation to metabolic functions essential to biotechnology/system biology remain scarce. We address this gap by building a pangenome-wide gene-protein-reaction association and applying it to 2377 *Escherichia coli* genomes to reconstruct a pangenome-scale metabolic model (panGEM). We validate panGEM against Biolog carbon source utilization assays, achieving ≈0.99 precision in growth/no-growth predictions. Using panGEM, we identify >11,000 rare metabolic genes, yet only 35 metabolic reactions are rare. To explain the mismatch, we examined rare genes and found that most are pseudogenes or diverged orthologs acquired by horizontal gene transfer (HGT). Results indicate a recurrent loss-reacquisition cycle in which a core allele is lost/pseudogenized and its function is restored by HGT, preserving function without expanding the reactome, generating genetic heterogeneity in a small subset (~3.6%) of reactions, marking selection pressure hotspots of metabolism. Thus, pangenome annotation reveals the evolutionary dynamics that shape the genetic basis of metabolism.

## Teaser

Bacterial pangenome annotation reveals loss-reacquisition cycles shaping metabolism.

## INTRODUCTION

The increased affordability of whole-genome sequencing in the early 2010s led to the sequencing of tens of thousands of bacterial genomes and their subsequent public availability. These genomes opened up new avenues for comparative genomics at the pangenome scale for a bacterial species (*1*, *2*). Pangenomics has since become a novel addition to phylogenetics, enabling comprehensive analysis of taxonomic genotypes (*3*, *4*). Despite this wealth of genome sequencing data, linking genotype to phenotype across pangenomes remains a persistent challenge.

Shortly after the first whole-genome sequences were completed (*5*–*7*), functional annotations of the identified genes were performed. Of the 4600 genes found on the *Escherichia coli* K-12 MG1655 genome (*6*), about 3000 were functionally annotated, with about half of those being metabolic genes (*8*, *9*). Chemical equations could be associated with gene-encoding enzymes. These associations, when assembled at the genome scale, could be used to reconstruct genome-scale metabolic models (GEMs). The reconstruction was genome specific, where the metabolic reactions were tied to the encoding genes through gene-to-protein-to-reaction (GPR) associations (*10*, *11*). Metabolic network reconstructions provided the foundation for computational GEMs that formally link metabolic genotypes to phenotypes (*12*–*16*). Thus, genome-scale mathematical models of metabolic genotype-phenotype relationships were born and became foundational to system biology.

The pangenome of a species is the set of all nonredundant genes observed across its strains; it comprises the core genome (genes present in nearly all strains), the accessory genome (genes present at intermediate frequencies), and rare genes (low-frequency genes, including singletons) (*1*, *17*). In practice, a pangenome is built by collecting many genomes of a species, normalizing/updating their annotations, grouping similar genes into families (clustering at a defined sequence similarity cutoff), and forming a presence-absence matrix that records which genomes carry which gene cluster (*18*–*20*). Gene families then can be classified as core, accessory, or rare based on how frequently they occur (*1*, *17*). This framework can be used to quantify genome fluidity (open versus closed pangenomes), track horizontal gene transfer (HGT) and gene loss, study evolutionary dynamics, relate gene content to ecology and host adaptation, identify marker genes—including resistome and virulence/pathogenicity determinants—prioritize targets for biotechnology (e.g., strain engineering and pathway design), and refine taxonomy and strain typing (*21*–*24*).

Pseudogenization and HGT are the primary forces that diversify bacterial pangenomes (*25*). Ongoing mutation and deletion continually generate pseudogenes, most of which persist only briefly before further decay or removal; bursts of pseudogenes are especially common in lineages undergoing niche shifts (e.g., host association) and contribute to between-strain gene content differences (*25*) In parallel, HGT via mobile genetic elements introduces novel alleles and functions, supplying adaptive traits that spread within populations and inflate the accessory and rare gene pools (*20*). Together, recurrent gene loss and gene gain shape the characteristic core-accessory-rare structure of pangenomes observed across bacteria, including the “open” pangenomes first quantified for pathogens such as *Streptococcus agalactiae* (*1*). Recent theoretical and empirical work further shows that gene content dynamics reflect a balance of persistent (core-like) genes with private and mobile fractions, consistent with models in which HGT and differential loss drive much of the observed diversity (*26*). Last, for metabolic genes, HGT can yield homologous replacements of lost functions or, more rarely, nonhomologous isofunctional enzymes, complicating pathway inference and reinforcing the need for pangenome-aware analyses (*27*).

The *E. coli* pangenome is considered moderately open, with considerable genetic diversity across strains expanding as new genomes are sequenced, revealing substantial variability among its phylogroups (*28*). The *E. coli* core genome closes at around 2398 genes, and the accessory genome reaches approximately 5182 genes, but the number of novel gene clusters steadily increases with new sequenced genomes, driven by genes that are found in single or a handful of strains (rare genes) (*29*). These rare genes are expected to reflect unique adaptations, allowing *E. coli* strains to thrive in diverse ecological niches (*30*).

We can now address the metabolic genotype-phenotype challenge at the pangenome scale. We can formulate pangenome-wide collections of GPRs (panGPRs) from a large number of sequenced strains of a species, effectively functionally annotating the pangenome. In this study, we show that the panGPRs reveal multiple different genetic bases for a total of 2753 metabolic reactions in *E. coli*. This genetic variation reflects various adaptive genetic mechanisms. Whereas functional annotation of a genome reflects molecular mechanisms (proximal causation), we now have annotation of the pangenome that reflects genetic mechanisms (distal causation). These findings provide a quantitative framework for understanding bacterial metabolic evolution and offer critical insights for metabolic engineering, strain design, and therapeutic targeting in this model organism.

## RESULTS

### Pangenome, panGPR, and panGEM reconstruction

**Fig. 1.**
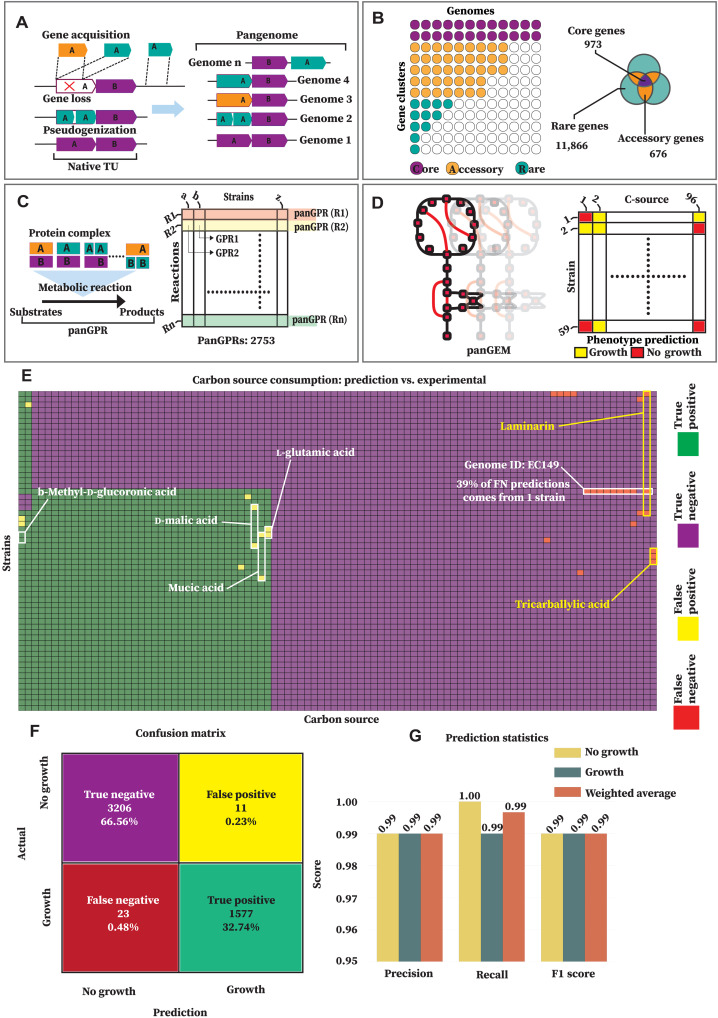
Reconstruction and validation of panGPRs and panGEM. (**A** to **D**) Formulation of panGPRs and panGEM. Genomes undergo pseudogenization and HGT, generating a pangenome in which metabolic gene clusters are classified as core, accessory, or rare, with most rare clusters representing pseudogenized core/accessory genes. All metabolic clusters were mapped to reactions by bidirectional BLAST against *Enterobacteriaceae* GEMs in the BiGG database to derive panGPRs, which were overlaid onto the *E. coli* pangenome to quantify the contribution of each gene class to every reaction. Individual genomes were then mapped to this pan-reactome to draft strain-specific networks, which were semiautomatically and manually. Subsequently, the panGEM was validated by reconstructing GEMs for 59 in-house strains and comparing predicted growth on 96 carbon sources with BioLog phenotypes. TU, transcriptional unit. (**E**) Comparison of predicted and experimental carbon source use. Heatmap of 59 strains (rows) on 96 compounds (columns) (see fig. S5 for details). Colors indicate agreement or disagreement between model and BioLog data; most false negatives cluster in a single strain (EC149). (**F**) Carbon source confusion matrix. Counts and percentages of true negatives, false negatives, true positives, and false positives summarizing prediction performance. (**G**) Prediction statistics. Bar plot of precision, recall, and *F*_1_ score derived from the validation results.

We use the pangenome to study how metabolic pathways evolve. Pseudogenization, loss, and reacquisition reshape coding sequences across strains and generate the sequence diversity that defines the pangenome. These processes actively drive bacterial adaptation and evolution (Fig. 1, A to D). We built a pangenome for *E. coli* from a set of 2337 completely sequenced genomes (see data S1 for a full genomes list). Only genomes designated as “complete” were included, to minimize technical artifacts—such as missing or pseudogenes at contig termini—arising from genome assembly contiguity. A pangenome groups orthologous alleles into gene clusters using a sequence identity threshold, so the choice of threshold must reflect the biological question. We aimed to cluster orthologs within *E. coli* species and to treat splits between clusters as meaningful divergence rather than routine allelic drift. At the whole-genome level, species are delineated at ~95 to 96% average nucleotide identity (ANI) (*31*). By contrast, between-genus comparisons inside *Enterobacteriaceae* fall far lower: for example, *E. coli* versus *Salmonella enterica* is ~84% ANI, a canonical cross-genus, same-family pair (*32*). Guided by this scale, we used 80% nucleotide identity in Cluster Database at High Identity with Tolerance (CD-HIT): Clusters separated by >20% divergence are conservatively beyond ordinary intraspecies allelic variation and approach the deep divergence typical of cross-genus contrasts in this family, making them biologically meaningful units for downstream assessment (see fig. S1A for sensitivity analysis on the similarity threshold).

The gene clusters in the pangenome then can be classified into three categories based on their prevalence [see (*33*)]: (i) the core genome, containing gene clusters found in the vast majority of strains; (ii) the accessory genome, containing gene clusters found in some strains; and (iii) the rare genome that contains gene clusters found in a few strains. We defined cluster prevalence as the fraction of genomes carrying a given gene cluster (0 to 100%). To set nonarbitrary bins, we constructed the empirical cumulative distribution of cluster prevalence across all metabolic clusters, fit a smooth logistic curve, and located the inflection point (zero of the second derivative) (*34*). Using the S-shaped profile, we then measured arc-length from the inflection point to each endpoint and defined core as clusters within the upper 90% of that distance toward 100% prevalence (the “upper elbow”) and rare as clusters within the lower 90% of the distance toward 0% prevalence (the “lower elbow”); accessory comprised the remainder. This yielded stable, data-driven thresholds: core of >96.7%, accessory of 6.8 to 96.7%, and rare of <6.8% (Fig. 1B) [see fig. S2; also see fig. S1 (B to G) for sensitivity analysis on the prevalence threshold]. As with these gene clusters, metabolic reactions can be classified similarly into core reactions, accessory reactions, and rare reactions. Thus, relationships between metabolic gene clusters and reactions can be studied at the pangenome scale using GPRs.

### Computing metabolic phenotypes from panGPRs with a high degree of accuracy

We generated panGPRs, by compiling *Enterobacteriaceae* GPRs from the BiGG database (http://bigg.ucsd.edu/) (see data S2 for a full list of template reconstructions) and mapped them to the *E. coli* pangenome, yielding a panGPR set covering 2753 reactions and linking 13,515 metabolic gene clusters to those reactions. The completeness and accuracy of the GPRs for a strain is assessed by converting the metabolic network reconstruction into a computational genome-scale model of metabolism (*13*). A pangenome-scale metabolic model (panGEM) of *E. coli* metabolism was then constructed by mapping the complete set of panGPRs onto the 2377 fully sequenced genomes, yielding a panGEM across different phylogroups of *E. coli* (fig. S3, A and B). To assess the quality of the panGPRs, an additional set of GEMs was built for a set of 59 fully sequenced and phylogroup-balanced in-house *E. coli* strains (fig. S4, A and B). These 59 GEMs were used to predict metabolic (growth/no growth) phenotypes that were compared to measured phenotypes (Fig. 1D). The metabolic phenotypes were measured using BioLog plates where 16,992 growth conditions were generated in replicates (16,992 = 59 strains *96 wells *3 replicates) (Fig. 1E) (also see fig. S5).

The confusion matrix (Fig. 1F) provides a detailed breakdown of the panGEM’s prediction performance. True negatives (3206 instances, 66.56%) are correctly identified as “no growth” conditions, while true positives (1577 instances, 32.74%) are correctly identified as “growth” conditions. False positives (11 instances, 0.23%), predict growth where none is observed, and false negatives (23 instances, 0.48%) predict no growth where growth is observed. The combined results indicate a very low error rate in the panGEM’s predictions.

Additional prediction statistics further underscore the panGEM’s robustness, with precision, recall, and *F*_1_ score metrics all averaging around 0.99 (Fig. 1G). This result suggests that the panGEM not only accurately predicts growth and no growth conditions but does so consistently across different strains. The high precision indicates that the panGEM’s predictions for growth are mostly correct, while the high recall ensures that most of the actual growth conditions are identified. The *F*_1_ score, which balances precision and recall, confirms the overall effectiveness of the panGEM.

This large-scale evaluation demonstrates panGEM’s predictive capabilities. Its high accuracy indicates that the quality of (i) the genomic sequences, (ii) their annotation, (iii) the assembled panGPRs, and (iv) the metabolic network reconstruction is high, leading to reliable predictions.

### Open pangenome, closed pan-reactome: Pseudogenization and xenologs dominate rare clusters

**Fig. 2.**
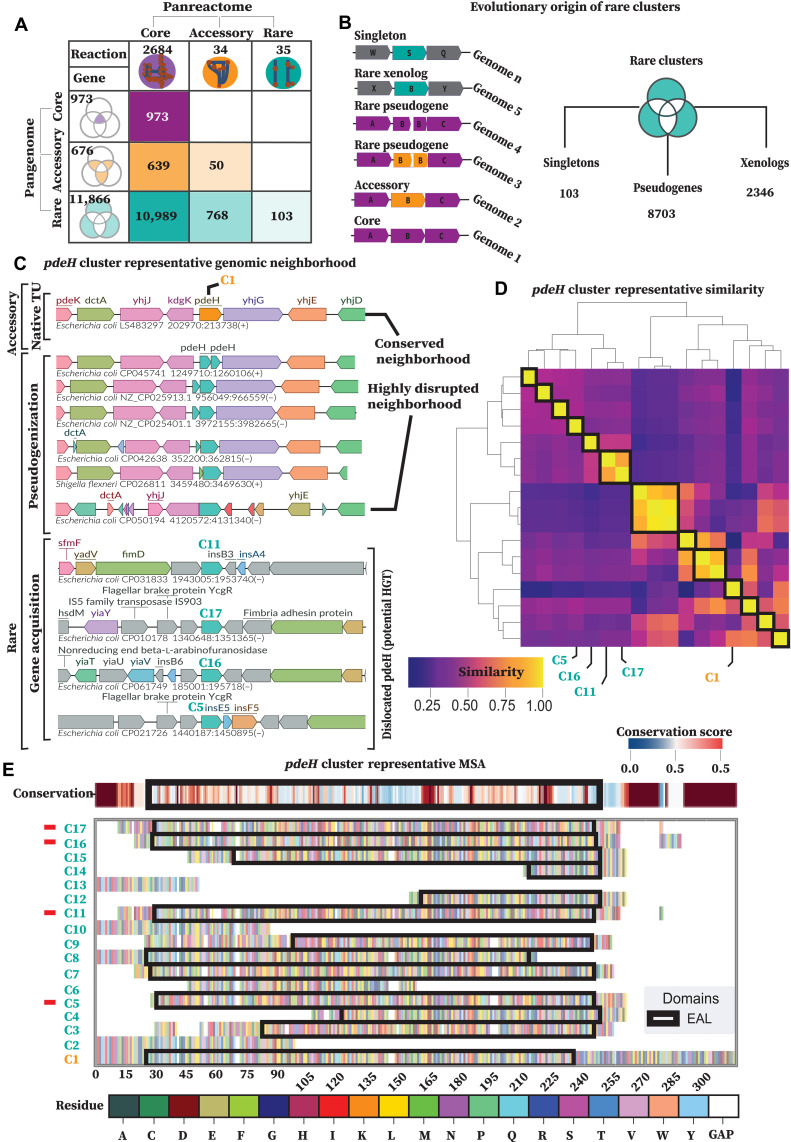
Rare gene classification. (**A**) Illustration of the flow of genes from different categories of pangenome (core, accessory, and rare) to reaction categories (core, accessory, and rare) while core genome only encodes core reactions, a large fraction of rare and accessory genomes also encodes core reactions, leading to an open pangenome but closed reactome. (**B**) Rare gene clusters are classified into singletons, pseudogenes, and diverged horizontally transferred orthologs (xenologs) genes. Singleton genes encode rare metabolic reactions, while xenologous genes encode reactions that core and accessory genes could also encode. (**C**) The reference genome neighborhood in the first row represents an accessory cluster encoding PdeH. The other neighborhoods show rare clusters, including pseudogenized and intact rare clusters (C5, C11, C16, and C17), with pseudogenized genes similar to the reference, while intact clusters are in different genome neighborhoods, indicating possible HGT events. (**D**) The heatmap shows DNA sequence similarity among representative alleles from 17 clusters encoding the *pdeH* gene. Seventeen clusters encode *pdeH* with at least 20% nucleotide divergence. The heatmap clusters are based on amino acid similarity, forming 13 protein clusters, each with at least 20% amino acid divergence. (**E**) Multiple protein sequence alignment (MSA) of *pdeH* clusters based on translated protein sequences. Representative alleles from distinct clusters (C1, C2, etc.) are shown, with text colors indicating pangenome category (teal: rare, orange: accessory). Gene clusters likely introduced by HGT are indicated by a red line behind their cluster name. The first row shows codon conservation across clusters, from low (blue) to high (red). Pfam functional domains are shown in black boxes.

Of the 13,515 metabolic gene clusters forming the panGPRs, 11,866 (87.8%) are rare, 676 (5.0%) are accessory, and 973 (7.2%) are core (Fig. 2A). Despite this abundance of rare clusters which is an indicator of open pangenome, the pan-reactome appears closed. Reaction presence-absence across strains yields 2684 core, 34 accessory, and 35 rare reactions (Fig. 2A). This mismatch—many rare gene clusters but few rare reactions—indicates that most rare clusters either encode existing reactions or are nonfunctional pseudogenes, rather than introducing new biochemistry. We therefore analyzed the rare metabolic gene clusters in detail to understand their role in metabolism of *E. coli* as a species.

We define rare genes in three categories: (i) pseudogenes: Most rare gene sequences are pseudogenized versions of core or accessory genes. These pseudogenes result from premature stop codons that break a core/accessory gene into smaller sequences. These pseudogenes form separate clusters and are classified as rare because such gene breakages are infrequent. (ii) Singletons: These are intact, functional genes encoding rare metabolic enzymes. They represent true rare genes, as they introduce additional functions to the bacterial metabolism, providing enzymes not found in most strains within the species. (iii) Horizontally transferred orthologs (xenologs): These are intact but diverged genes that encode enzymes also found in core or accessory gene clusters (Fig. 2B). Xenologs show extensive divergence, with less than 50% similarity to the core sequences encoding the same reactions. These genes are located in different genomic neighborhoods compared to their core/accessory counterparts. Of the 11,866 rare metabolic gene clusters, 8703 (73.3%) are pseudogenes (nonfunctional pseudogenes), 3055 (25.7%) are xenologs (intact, HGT-displaced orthologs of existing reactions), and 108 (0.9%) are singletons (intact genes introducing new reactions relative to the core and accessory repertoire). Thus, 73.3% of rare clusters are nonfunctional, and only 108 of 11,866 = 0.91% expand the reactome. Among functional rare clusters (xenologs + singletons = 3163), xenologs account for 96.6% and singletons for 3.4% (Fig. 2B), indicating that rare-cluster diversity translates into plasticity of the genetic basis of core reactions rather than introducing additional biochemistry and expanding pan-reactome, an observation consistent with a closed pan-reactome.

### The same metabolic reaction can be catalyzed by divergent gene clusters

To illustrate why rare clusters expand genetic diversity without expanding the reaction set, we examined *pdeH*, a cyclic diguanosine monophosphate (c-di-GMP) phosphodiesterase gene neighborhood (Fig. 2C), protein similarity (Fig. 2D), and functional domains (Fig. 2E). Nucleotide sequences formed 17 clusters: one prevalent accessory cluster (C1 found in 2234 genomes) and 16 rare clusters. Genome-neighborhood analysis placed C1 in a conserved operon, whereas many rare clusters were pseudogenes within that locus or intact but relocated to distant loci, frequently adjacent to insertion sequences or transposons—consistent with HGT-mediated displacement (Fig. 2C).

Translation and reclustering at 80% amino acid identity reduced the 17 nucleotide clusters to 13 protein clusters: eight pseudogenes, four intact, relocated xenologs (C5, C11, C16, and C17), and the reference C1 (Fig. 2D). Despite ~25% identity to C1, the intact, relocated clusters retained hallmark EAL phosphodiesterase features. EAL denotes the conserved Glu-Ala-Leu motif in the c-di-GMP phosphodiesterase domain that hydrolyzes c-di-GMP to pGpG via divalent metal–dependent catalysis. The presence of this motif and surrounding catalytic residues indicates xenologous replacement of the same reaction. In contrast, several pseudogene clusters lacked part or all of the EAL catalytic domain, indicating loss of function (Fig. 2E).

### The genetic basis of about 100 metabolic reactions is remarkably diverse

**Fig. 3.**
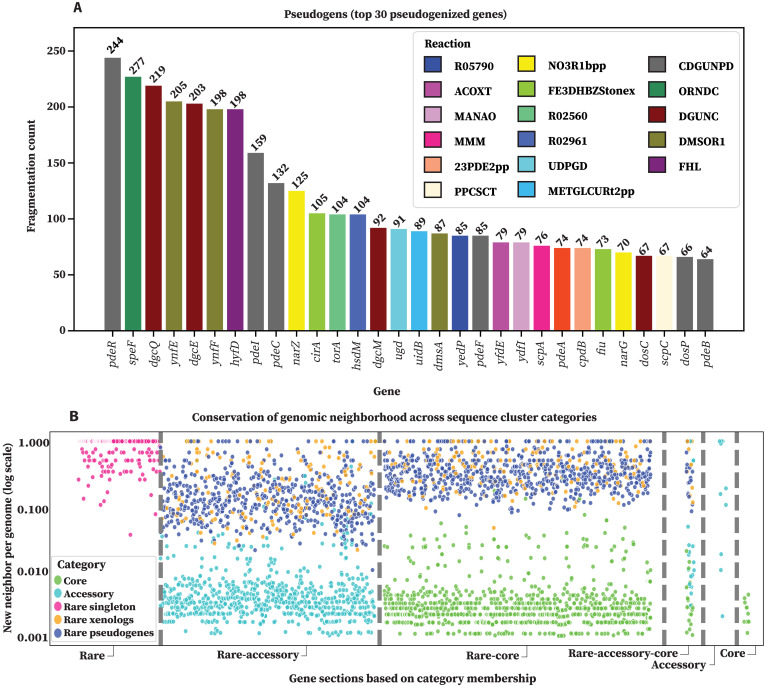
Pseudogenization and HGT at scale. (**A**) Pseudogenization events across metabolic pathways. All panGPRs were analyzed to identify pseudogenes and the reactions they encode. Pseudogenes genes are shown on the *x* axis, with their count across genomes on the *y* axis. Bars are color-coded on the basis of the reactions these genes encode (the definitions of the abbreviated names of the reactions are found in the BiGG database). (**B**) Genomic neighborhood analysis. Genes are grouped on the *x* axis (rare, rare-accessory, rare-accessory-core, rare-core, accessory, and core) based on the categories of clusters that can encode them. The conservation of the genomic neighborhood is shown on the *y* axis, based on the number of new neighbors (relative to the dominant neighborhood) found for each gene per genome (value of 1 indicates a new neighbor per genome thus low conservation) (see Materials and Methods). The term “neighbor” only refers to immediate genes up and downstream the target gene (only one gene distance). Genes are color-coded on the basis of their pangenome category.

We extended the *pdeH* analysis to all metabolic reactions by evaluating the panGPR matrix across 2753 reactions. Two global patterns emerged. First, pseudogene distribution was uneven across pathways (Fig. 3A): Genes associated with CDGUNPD isozymes (*pdeR*, *pdeI*, and *pdeC*), diguanylate cyclases (*dgcQ* and *dgcE*), *speF*, selenate reductase subunits (*ynfE* and *ynfF*), and *hyfD* showed the highest frequencies (Fig. 2C). Second, genomic neighborhoods distinguished categories: Core/accessory clusters were syntenically conserved, whereas rare clusters—xenologs and pseudogenes—occurred in divergent loci frequently flanked by insertion sequences or transposases (Fig. 3B). These genome-wide signals explain high genetic diversity without commensurate reaction gain.

**Fig. 4.**
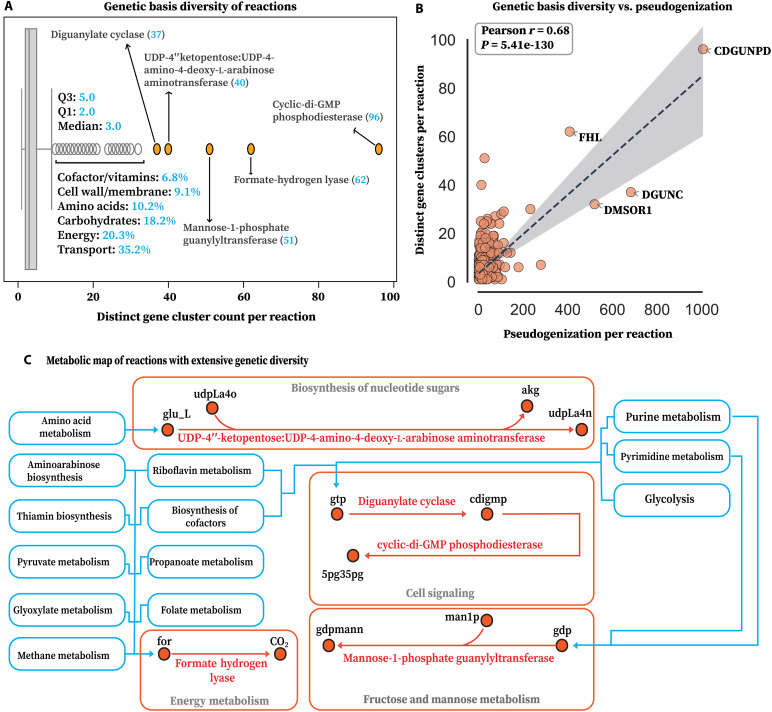
Diversity in the genetic basis of *E. coli* metabolism. (**A**) The boxplot shows the distribution of distinct gene clusters encoding each reaction (genetic diversity of panGPRs), with rare pseudogene clusters excluded (only functional genes are considered). Outliers represent reactions with extended genetic diversity. The annotated median indicates the point where 50% of reactions lie. (**B**) Correlation between pseudogenization and the diversity of the genetic basis of reactions. Dots represent reactions. The *x* axis shows the number of pseudogenization events, while the *y* axis shows the number of distinct gene clusters encoding each reaction across the pangenome (excluding pseudogenes). A Pearson correlation coefficient of 0.61 indicates a moderate to strong correlation between pseudogenization events and the diversity of the genetic basis of reactions. (**C**) Metabolic map of reactions with extensive diversity in their genetic basis. udpLa4o, UDP-4-keto-pyranose; glu_L, l-glutamate; akg, 2-oxoglutarate; udpLa4n, uridine 5′-diphospho-β-4-deoxy-4-amino-l-arabinose; gtp, GTP; for, formate; cdigmp, cyclic diguanosine monophosphate; 5pg35pg, 5-phosphoguanylyl(3->5)guanosine; gdpmann, guanosine diphosphate (GDP)–d-mannose; man1p, d-mannose 1-phosphate; gdp, GDP.

After excluding pseudogenes, we counted intact clusters per reaction. Twenty-five percent (25%) of reactions were encoded by ≥5 clusters (Fig. 4A). Across genes, pseudogene frequency correlated with allelic diversity per reaction (*r* = 0.61; Fig. 4B). Approximately 100 reactions were outliers with >30 clusters, concentrated in six metabolic subsystems including transporters, energy metabolism, carbohydrate catabolism, amino acid biosynthesis, cell wall/membrane biosynthesis, and cofactor/vitamin metabolism (Fig. 4A); the observed genetic diversity of these reactions is robust against pangenome similarity threshold (fig. S1H). Reactions with the highest count of gene clusters were CDGUNPD and diguanylate cyclase (fig. S6) (DGUNC), selenium containing formate hydrogenlyase (FHL), mannose-1-phosphate guanylyltransferase, and UDP-4″-ketopentose: UDP-4-amino-4-deoxy-l-arabinose aminotransferase (Fig. 4C). Diversity within FHL localized to HyfD subunit. Overall, reaction-level diversity was concentrated, while the remainder of the reactome remained genetically conserved. One can thus hypothesize that these metabolic processes are under environment-specific selection pressure that has resulted in their loss and recovery in the evolutionary history of these strains

### Many strains have rare genes associated with aromatic amino acid and branched-chain amino acid biosynthesis

The tryptophan biosynthesis pathway was found to be enriched in rare gene clusters. Rare clusters were detected for all three isozymes of 3-deoxy-d-arabino-heptulosonate-7-phosphate synthetase (DDPA). DDPA catalyzes the first commitment step in aromatic amino acid biosynthesis. This multimeric enzyme complex has 13 rare *aroH* alleles across 41 strains, 9 rare *aroG* alleles (fig. S8) across 19 strains, and 10 rare *aroF* alleles (fig. S7) across 25 strains. Whereas *aroG* and *aroF* each retain a core gene and lack accessory copies, *aroH* is encoded solely by three distinct accessory clusters, with no core copy present. This distribution of genes demonstrates that an essential enzymatic function can be maintained without a conserved core gene, being instead fulfilled by sequence-divergent rare and accessory genes across different strains.

**Fig. 5.**
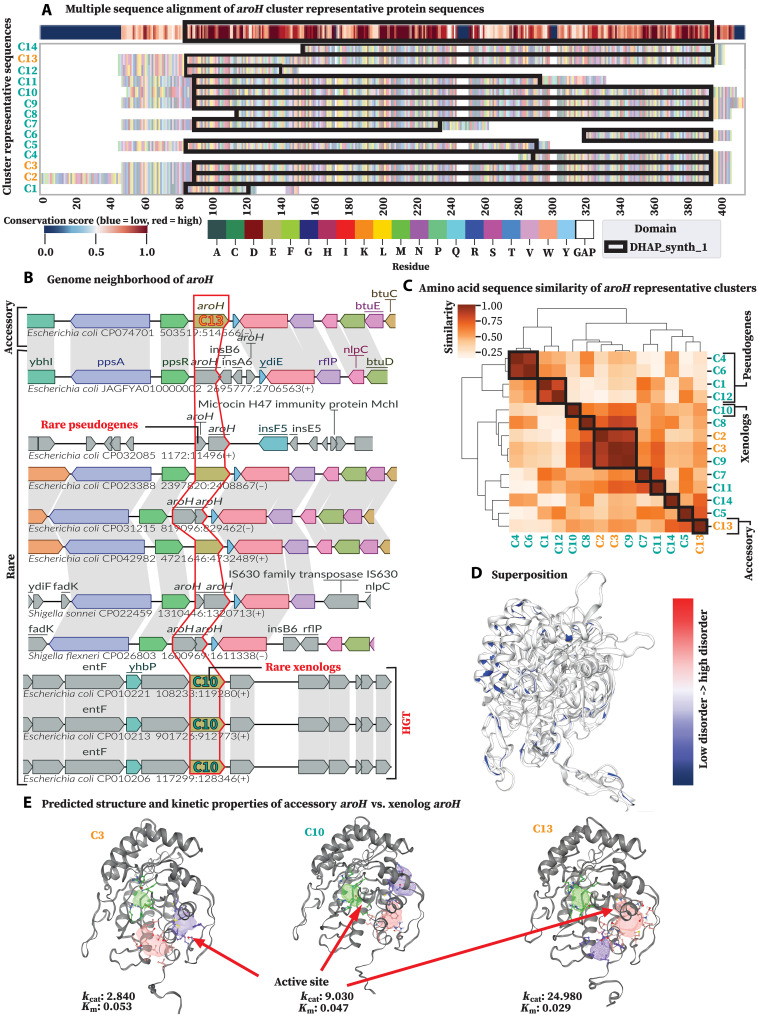
*aroH* multiple gene sequence alignment and its genomic neighborhood. (**A**) MSA of rare *aroH* genes to accessory *aroH* gene. Alignment is across the 415 codons in the gene. Representative alleles from each distinct cluster identified by cd-hit (80% nucleotide similarity) are presented numerically (C1, C2, etc.), text colors show the pangenome category of each cluster (teal: rare, orange: accessory). Colors represent amino acids, and white space represents gaps, the first row represents conservation of each codon across all clusters coded from blue (low conservation) to red (high conservation). Functional domains identified by pfam are shown in black boxes on the MSA plot. (**B**) Genomic neighborhood of *aroH* genes separated by cluster categories (accessory and rare). Gray shading connects homologous genes across the genome neighborhoods. The anchor gene, *aroH*, is shown in brown and highlighted by a red box. The reference genomic neighborhood is shown at the top of the plot representing an accessory cluster that is the most dominant gene cluster encoding *aroH* across the pangenome. Some of the rare genes have a similar genomic neighborhood as the reference, but some are highly different, as those annotated as possible HGT events with an entirely different neighborhood compared to dominant neighborhood. (**C**) Heatmap shows the similarity of representative alleles from each cluster encoding *aroH* at protein level. (**D**) Three-dimensional (3D) protein structure modeling and alignment: The 3D models of protein structures were generated for the reference gene (C13), a rare horizontally transferred gene (C10), and a less frequent accessory cluster (C3) with considerable divergence compared to C13. The 3D alignment compares structures of selected clusters against the reference gene, and structural divergence is color-coded from red to blue (high disorder–low disorder). (**E**) 3D models of selected sequences with predicted active sites and predicted kinetic properties (*K*_m_ and *k*_cat_).

Multiple sequence alignment of 13 *aroH* gene clusters (three accessory clusters: C2, C3, and C13; 10 rare clusters) was performed (Fig. 5A) followed by gene neighborhood analysis (Fig. 5B) and assessment of functional domains (Fig. 5C). The alignment indicates that most rare clusters are derived from gene pseudogenization and thus represent rare pseudogenes; nine of these arise from pseudogenization of accessory clusters. One rare cluster, C10, remains intact yet exhibits ≥55% divergence relative to all other clusters, including the accessory set (Fig. 5C). The central core of the DHAP synthase domain remains highly conserved across all clusters, but the N-terminal and C-terminal regions diverge by as much as 60% (Fig. 5A), indicating that C10 represents a xenologous variant of *aroH*.

Genomic neighborhood analysis further confirms C10 as a xenologous rare cluster, as C10 is found in a region entirely distinct from the native transcriptional unit (Fig. 5B). By contrast, the rare pseudogenes remain within the native transcriptional unit, whose up- and downstream regions are frequently disrupted by insertion sequences and transposable elements (*insA6*, *insB6*, *insC6*, *insD6*, *insE5*, *insF5*, and IS630 family transposases).

The diversity of the genetic basis of *aroH* was expected to alter the three-dimensional conformation of the DDPA enzyme and thus affect its catalytic kinetics. To evaluate this difference, homology models of two accessory variants (C3 and C13) and the xenologous rare variant (C10) were generated, each achieving a confidence score of 99%. The models were then superimposed to detect regions of structural disorder relative to the consensus fold (Fig. 5D). Despite substantial sequence divergence, no major backbone disorder was observed across any model; however, localized increases in flexibility were detected at specific loop regions—elements known to modulate enzyme kinetics.

From these structural models, Michaelis-Menten parameters were predicted (*35*). The predominant accessory variant, C13, was found to exhibit the highest *k*_cat_ and lowest *K*_m_, whereas the less prevalent accessory variant, C3, showed reduced catalytic efficiency (lower *k*_cat_ and higher *K*_m_). The rare xenologous variant, C10, displayed intermediate values of both *k*_cat_ and *K*_m_, consistent with its sequence divergence and dislocated genomic context (Fig. 5E).

Similar genetic diversity is found in genes that encode enzymes in other aromatic amino acid biosynthetic pathways as well as those in branched-chain amino acid synthesis (figs. S8 and S9). We note that these amino acids are commonly auxotrophic (*36*), lending credence to a gene loss (under specialization) followed by gene acquisition (once strain leaves the specialized microniche) mechanism.

## DISCUSSION

We present a pangenome study of *E. coli* metabolism. Each strain genome is the outcome of a natural evolutionary experiment; aggregating thousands of these outcomes enables species-scale links from gene content to phenotype. Genome-scale metabolic reconstructions encode GPR rules that specify how genes map to enzymes and reactions using Boolean logic, providing a formal bridge from genotype to phenotype (*14*). This framework is computable and verifiable: Early studies reconstructed the *E. coli* network and prospectively compared in silico growth predictions to measurements, demonstrating quantitative agreement between the model and the experiment (*9*). Building on this foundation, multistrain modeling showed that strain-specific GEMs can explain species-level metabolic diversity and enable systematic phenotype prediction across conditions (*37*).

Here, we introduce panGPRs—the pangenome-wide collection of GPRs—as a species-level representation of the genetic basis of metabolism and a scaffold for quantitative tests of metabolic function. From panGPRs, we reconstruct panGEMs and simulate growth across media to generate falsifiable predictions. In our evaluation against high-throughput Biolog assays (>16,000 strain condition phenotypes), panGPR-derived GEMs achieved ~99% precision and ~99% accuracy for growth calls, verifying that these reconstructions recover phenotypic consequences of gene content variation at scale. Together, panGPRs and panGEMs provide powerful tools to study the genetic basis of metabolism that are quantifiable and verifiable.

All growth predictions use the *i*ML1515 biomass objective. *E. coli* strains differ not only in biomass stoichiometry but also in composition itself—O-antigen biosynthesis spans >180 serogroups, each requiring unique sugar precursors [e.g., perosamine, colitose, and guanosine diphosphate (GDP) fucose] absent from K-12 metabolism, and capsular polysaccharides such as the K1 antigen demand CMP-Neu5Ac, a precursor not represented in *i*ML1515. However, these strain variable surface components are dispensable for growth under standard laboratory conditions. For binary growth/no-growth predictions, what matters is whether the network can produce the conserved core biomass precursors such as amino acids, nucleotides, lipids, and peptidoglycan, which are highly conserved across *E. coli* (*16*, *38*). Strain-specific biomass equations remain a valuable future direction, particularly for quantitative flux predictions, where objective function sensitivity is more pronounced.

Using the panGPR, we find that genetic conservation holds for most reactions, yet ~100 reactions deviate: A minority of strains encodes the same function with rare genes instead of the core gene. This replacement preserves enzymatic capacity while introducing genetic heterogeneity, an observation consistent with models in which recurrent gene loss and gain shape pangenomes and maintain an open accessory gene pool (*22*). We trace this heterogeneity to two classes of rare metabolic gene clusters: (i) pseudogenes (mostly products of core/accessory gene decay and truncation) (*38*); (ii) diverged horizontally transferred orthologs, also referred to as xenologs (*39*–*41*).

Pseudogenes reflect ongoing pseudogenization of core or accessory genes; pseudogenization creates auxotrophies by breaking functional genes. This process is common during niche shifts and host adaptation, where relaxed selection on specific pathways accelerates gene decay (*42*, *43*). A global analysis of >26,000 bacterial genomes shows that amino acid auxotrophies are unevenly distributed, with branched-chain auxotrophies among the most frequent and aromatic auxotrophies also common, consistent with pathway-specific vulnerability to loss (*44*). These patterns match our observations in *E. coli*: Pseudogene-driven breaks in branched-chain and aromatic amino acid biosynthesis (fig. S8) coincide with documented auxotrophies, and reacquisition via HGT provides the most parsimonious explanation for restored function in strains that leave specialized niches. This loss-and-reacquisition cycle integrates two established trends: deletion bias and pseudogenization that reduce metabolic scope and targeted HGT that not only rebuilds specific pathways when environments change but also enhances the species biodiversity by introducing diverged orthologs into species pangenome.

Xenologs are orthologous genes whose histories include at least one horizontal transfer event (*39*). In our dataset, xenologs do not distribute evenly across pan-metabolic network. They concentrate in specific pathways and cellular processes, particularly in reactions that are involved in transition between planktonic and biofilm lifestyles. We also noticed that they do not co-occur with core alleles, which is consistent with replacement rather than encoding additional function (*22*). This pathway bias aligns with theory and comparative analyses showing that transferability varies by functional context and network topology, with higher mobility in certain metabolic modules and lower mobility where connectivity is high (*45*). Mechanistically, plasmids, integrative and conjugative elements, transposons, integrons, and phage mediate these events and leave recognizable signatures near insertion sites (*46*). Our findings reveal species-specific “plasticity hotspots” in the metabolic network in which reactions remain conserved while the genetic basis of reactions turn over via loss and reacquisition events. This helps explain strain-dependent essentiality and neighborhood rewiring (*3*), warning against single-allele therapeutics and motivating therapeutic target sets that account for genetic diversity.

In contrast, singletons are rare, intact genes that encode additional reactions not represented in the core or accessory genome (*38*); therefore, they are the only group of rare genes that encode rare reactions and introduce additional biochemistry into the strains that carry them. A large cross-species analysis defined a singleton metric (singleton intact and singleton pseudogene) and showed that rare intact genes, including singletons, can bear signals of selection, yet they form only a small fraction of total gene turnover across 668 species (*38*). Mapping the positions of singletons therefore shows where the metabolic network is expanding and highlights pathway-level plasticity that varies by species. Scarcity of singletons explains why the *E. coli* reactome appears closed with only 35 rare reactions despite many rare gene clusters. Most rare gene clusters are pseudogenes or xenologs rather than genes that add new reactions relative to the core and accessory repertoire.

The reactions with the highest genetic heterogeneity are involved in processes central to environmental transitions, such as cyclic-di-GMP signaling, the selenium-containing FHL complex, polysaccharide precursor biosynthesis, and lipid A modification that suggests ecology-dependent selection. Spatial omic studies of biofilms indicate regional activation of specific catabolic and redox modules (including c-di-GMP–linked signaling and hydrogen metabolism) (*47*), supporting the view that microenvironments within structured communities impose heterogeneous selection on metabolic circuitry (*47*). Although xenologs can compensate for lost core genes, in *E. coli* metabolism, we found that these xenologs are highly divergent and inserted into non-native genomic neighborhoods, and this sequence and positional variation quantitatively alters the rescued function, In this context, xenologs may have different expression levels than the core gene (*48*) and/or have different kinetics and flux even when reaction stoichiometry is conserved (*49*, *50*), potentially producing strain-specific metabolic phenotypes in niches such as hypoxic biofilm interiors versus planktonic growth.

We also observe that pseudogenization-prone pathways (e.g., branched-chain and aromatic amino acid biosynthesis) coincide with auxotrophies frequently reported in *E. coli* (*36*, *44*). A plausible mechanism is niche-specific gene loss (driven by relaxed selection) followed by HGT-mediated reacquisition upon ecological shift, restoring essential steps for survival; our results in these amino acid pathways are consistent with that loss-and-reacquisition pattern. More broadly, inspection of genomic neighborhoods shows that rare metabolic genes occupy highly variable loci and are often adjacent to transposable elements—markedly different from the conserved neighborhoods of the corresponding core/accessory genes—providing locus-level evidence for HGT in the metabolic gene set (*51*).

Our conclusions reflect the genomes available and how they were processed. We analyzed only complete assemblies to reduce contiguity artifacts, which limits sample size and likely underrepresents strain diversity found in environmental, agricultural, and geographically undersampled contexts; public repositories are enriched for clinical and enteric isolates, introducing bias. Absolute counts of rare/accessory/core clusters also depend on the chosen annotation stack, clustering cutoff (CD-HIT 80%), and the data-driven prevalence thresholds used to bin clusters. For broader applicability, future work should expand the genome panel with ecology- and geography-balanced sampling when more genomes are sequenced.

In sum, we advance the concept of pangenome annotation by mapping alleles, gene clusters, proteins, and reactions across thousands of genomes to identify where a species’ metabolism is genetically plastic. In *E. coli*, >11,000 rare metabolic clusters define an open metabolic pangenome, yet the reactome remains closed—only ~100 of 2753 reactions show high genetic variability, and these are concentrated in biologically interpretable processes. Thus, while conventional genome annotation emphasizes a molecular mechanism (proximal causation), pangenome annotation exposes the distal evolutionary mechanisms such as pseudogenization and HGT, which sculpt the genetic implementations of conserved metabolic functions. Together, these results align a species-wide genotype map with measurable phenotypes and provide testable hypotheses for how selection and gene flow reshape metabolism in natural environments.

## MATERIALS AND METHODS

### Genome selection and annotation

We collected *n* = 2377 complete *E. coli* genomes from the Bacterial and Viral Bioinformatics Resource Center (BV-BRC; 2021) and National Center for Biotechnology Information RefSeq, removing non–*E. coli* entries and plasmid-only records. We retained assemblies only if they were complete (*L*50 = 1, *N*50 > 4,000,000 bp) and passed quality filters (completeness > 98.1%, contamination < 3.1%). We deduplicated the merged set (including Mash-based redundancy filtering) to keep a single representative per duplicate cluster. All retained genomes were uniformly reannotated with PROKKA v1.14.5 (*52*) using consistent parameters to harmonize gene calls across sources. This complete-genome set was used for pangenome reconstruction and PanGEM generation.

### Pangenome generation

We reconstructed the *E. coli* pangenome by clustering all coding sequences with CD-HIT (cd-hit-est) using -c 0.80, -aL 0.80, and -n 5 (default for identity, ≥0.70) and -n 4 for exploratory runs with identity of <0.70. Clusters defined allelic families; we flagged paralogs when a genome contributed >1 allele to a cluster. We encoded the cluster presence/absence per genome to build a binary presence-absence (PA) matrix (rows = clusters, columns = genomes; present = 1, absent = 0). Core, accessory, and rare fractions were assigned by a cumulative gene distribution procedure (*29*): We constructed the cumulative distribution of cluster prevalence, located the inflection point of the S-shaped curve, and defined core as clusters within 90% of the distance from the inflection point to the upper endpoint (“elbow”), rare similarly from the inflection point to the lower endpoint, and accessory as the remainder; this yielded baseline thresholds core of >96.7%, accessory of 6.8 to 96.7%, and rare of <6.8%. For sensitivity, we repeated clustering over identities {65 to 95%} (plus <0.70 with -n 4), recomputed PA matrices, reapplied the cumulative distribution classification and alternative cutoffs (core, ≥{90 to 100}%; rare, ≤{1 to 10}%), and summarized total clusters.

### Reconstruction of panGPRs

PanGPRs were generated by mapping selected genomes to *Entrobacteriaceae* GPRs from the BiGG database (*53*). First, models were downloaded from BiGG models. Second, all GPRs were collected from the models, and discrepancies in reaction IDs and metabolite IDs were corrected to prevent errors in passing them to panGPRs. From mapping this curated GPR information, 67,054 alleles (strain-specific genes) lacking any BiGG annotation were subsequently queried against Kyoto Encyclopedia of Genes and Genomes (KEGG; https://www.genome.jp/kegg/), resulting in the identification of 30 reactions that had been absent from all BiGG models (see data S3). By integrating these 30 formulated reactions with those already present, a total of 2753 metabolic reactions were established. Each reaction was then associated with one or more gene clusters and thereby defining the panGPR. The final set of all panGPRs was called the panreactome containing 13,515 metabolic gene clusters forming the genetic basis of 2753 metabolic reactions. The Panreactome was later used as templates for ortholog calling during panGEM reconstruction.

### PanGEM reconstruction

The panreactome was used as a template for pangenome scale reconstruction of strain-specific GEMs. Panreactome gene sequences and biochemical information were fed into our previously generated pipeline (*37*) alongside reannotated target genomes, and the similarity threshold for ortholog calling was set to 90% for mapping genes of target genomes to GPR gene sequences using bidirectional BLAST. The output GEMs were then used to get a list of all locus tags in each model. These locus tags were then mapped against their respective genomes to find missed metabolic genes from their corresponding models. Missing genes were remapped to BiGG and KEGG databases using their annotation for finding their metabolic information. Genes with existing reactions in their corresponding models were added to their corresponding GPRs, while genes with nonexistent BiGG reactions underwent manual formulation of their reactions according to an available protocol (*14*) and were added to models. The biomass objective function was obtained from the *i*ML1515 model (*16*) and was added to all GEMs. GEMs corresponding to in-house genomes were used for validation of panGEM and then passed a gap-filling procedure on M9 media using our previous method (*37*). After gap filling, flux balance analysis was performed on M9 media to confirm the capability of generated models in producing biomass.

### Validation: Biolog PM data processing

We profiled 59 in-house *E. coli* strains on Biolog phenotype microarray plates PM01 to PM02 using M9 as the base medium. Precultures were grown in M9 + glucose (4 g liter^−1^) at 37°C with shaking and washed before inoculation to remove carryover carbon. Plates were prepared per the manufacturer’s instructions and run on an OmniLog for 48 hours at 37°C. Each strain was assayed in two biological replicates. For each well, time-resolved respiration curves were smoothed with a Savitzky-Golay filter. We extracted (i) maximum respiration, (ii) peak respiration rate, (iii) time to maximum, and (iv) area under the curve. For plates with a negative control well, we formed a control distribution from control well maxima and compared experimental wells via *z* or *t* tests to call significant metabolic activity. Plates with significant control signals were manually reviewed (contamination/background); after remediation, we reran tests and reinstated valid wells. Substrate utilization per strain-substrate pair was assigned from replicate consistency: 1 (used) if most of the replicates were positive, 0 (not used) if negative, and 0.5 (uncertain) if replicates lacked consensus. Uncertain calls and replicate discordant assays were excluded from the high-confidence evaluation set. An interactive browser for these data is available at https://pmkbase.com/species?specie=ecoli (*54*).

### Media simulation and computational growth prediction

We simulated growth in media matched to the Biolog conditions, with the base M9 minimal medium formulated taken from a previous study (*55*). All simulations used COBRApy v0.27.0 with the GLPK v5 linear solver; the biomass reaction was the objective in all optimizations using flux balance analysis. We classified no growth as a predicted growth rate <0.001 hour^−1^. Agreement between model predictions and Biolog calls was summarized with a confusion matrix (true negatives, false negatives, true positives, and false positives), and we reported accuracy and precision. To avoid inflating performance via reactions without gene associations, we excluded dipeptide substrates (e.g., Ala-Gly) because the required aminopeptidase steps are orphan reactions in the panGEM. Only assays with concordant biological replicates (growth or no growth consistent in all replicates) were retained for validation.

### Mapping GPRs to the pangenome

Genes of PanGEM GPRs were mapped to our reconstructed pangenome by their locus tags to identify the gene cluster of each locus tag. Subsequently, all gene clusters coding for the same reaction were identified and grouped on the basis of their reactions to reconstruct panGPR, which depicts the genetic basis of each reaction across the pangenome of *E. coli.*

### Gene genomic neighborhood analysis

The genomic location of each gene was extracted from GenBank files using Biopython to narrow down the visualization to the location of interest. For each gene of interest, genomes were selected to ensure that core, rare, and accessory variants of the target gene were represented in the chosen subset. LoVis4u version 0.0.10 (*56*) was used as the visualization tool to visualize the genomic neighborhood of target genes. Genome neighborhood conservation was assessed by extracting each target gene along with one flanking gene upstream and downstream. The number of new neighboring genes on either side was used as a measure of neighborhood instability. The number of new neighbors (relative to the dominant neighborhood) for each gene cluster was divided by the number of strains in which the cluster is present, yielding a conservation metric ranging from 0 to 1. A value of 1 indicates a different neighbor in every strain, reflecting complete neighborhood instability, whereas a value of 0 signifies no variation in neighboring genes across strains, indicating complete conservation.

### Multiple sequence alignment

For the comparison of rare, accessory, and core genes, we performed multiple protein sequence alignment (MSA) using Clustal Omega (*57*) and Biopython (*58*). In cases where core genes were available, they were used as the reference sequence to which rare and accessory genes were aligned. When core genes were absent, an accessory gene was selected as the reference. Gene sequences were obtained in FASTA format, with each gene’s alleles organized under their corresponding gene ID and locus tag. Sequences were aligned using Clustal Omega, and the longest representative sequence for each gene was selected for alignment.

### Prediction of 3D structure and 3D alignment and kinetic parameters

The three-dimensional (3D) protein structures were predicted using the AlphaFold server (*59*). Structural alignments were conducted using the structure assessment (*60*) server at swissmodel (https://swissmodel.expasy.org/assess).
