# Supplementary material for: Annotating the pangenome reveals the diversity in the genetic basis for metabolic enzymes
Source: Sci Adv. 2026 Jul 1;12(27):eaeb3363. doi: 10.1126/sciadv.aeb3363 (PMC13322260; doi:10.1126/sciadv.aeb3363)
Supplement: Supplementary file 1 — Figs. S1 to S9 Legends for data S1 to S3 [file sciadv.aeb3363_sm.pdf]

Science Advances
AAAS

Supplementary Materials for

Annotating the pangenome reveals the diversity in the genetic basis for metabolic enzymes

Omid Ardalani *et al.*

Corresponding author: Omid Ardalani, omidard@dtu.dk; Bernhard O. Palsson, palsson@ucsd.edu

Sci. Adv. **12**, eaeb3363 (2026)
DOI: 10.1126/sciadv.aeb3363

The PDF file includes:

Figs. S1 to S9
Legends for data S1 to S3

Other Supplementary Material for this manuscript includes the following:

Data S1 to S3

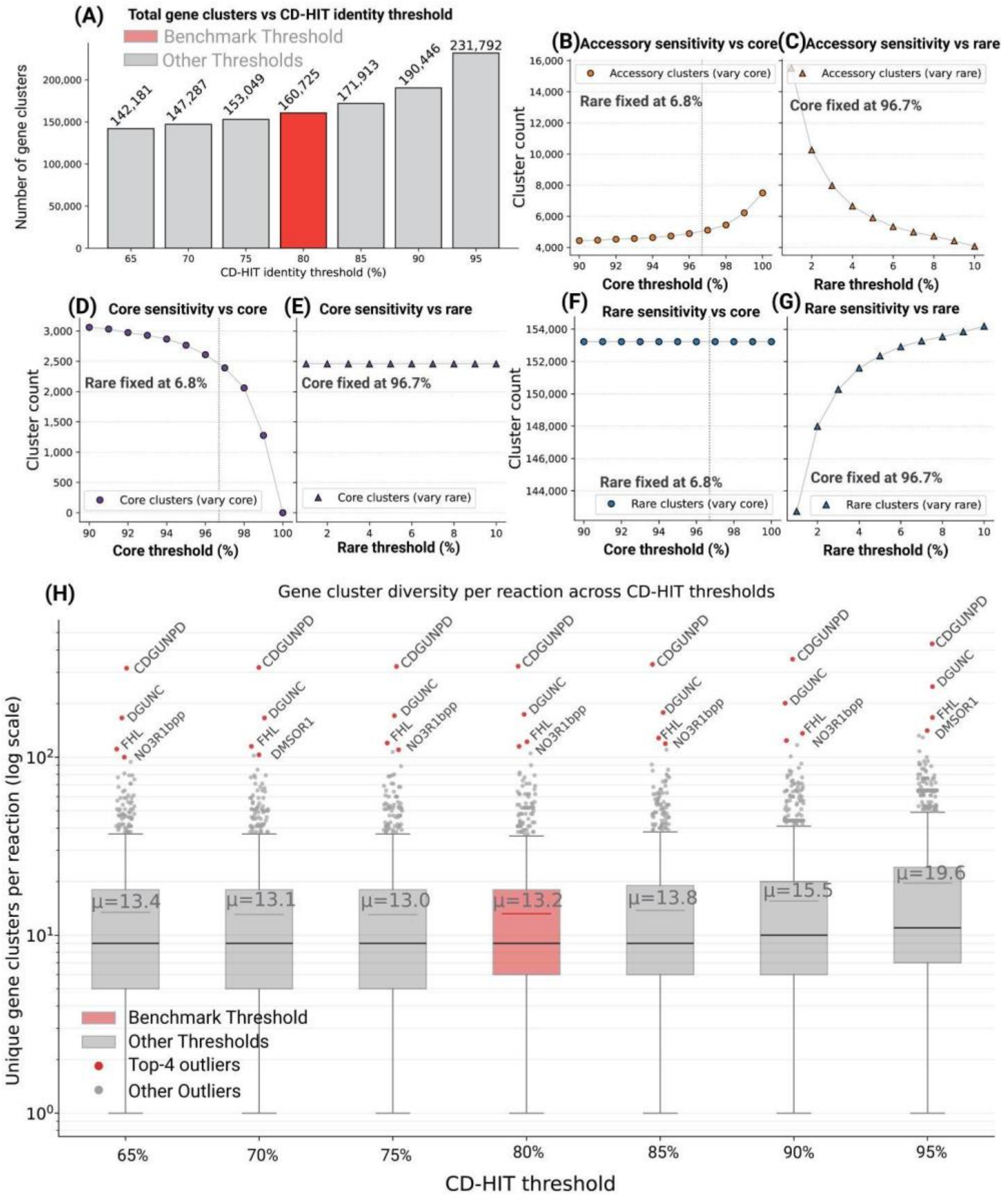

Fig. S1. Sensitivity of pangenome and panGPR results to clustering and prevalence thresholds. ***(A)*** *Total gene-cluster counts for seven pangenomes built with different CD-HIT sequence-identity thresholds. We selected 80% CD-HIT identity for the main analyses because it groups within-species allelic variants while separating highly diverged homologs; this is conservative relative to species demarcation (~95% ANI) and approximates genus-level divergence (~80% ANI).* ***(B–G)*** *Sensitivity of core, accessory, and rare cluster counts to prevalence cutoffs in the 80% CD-HIT pangenome, evaluated over a grid of core (high-prevalence) and rare (low-prevalence) thresholds.* ***(H)*** *Sensitivity of panGPR mappings to the CD-HIT identity threshold (e.g., number of reactions per gene cluster, reactions with multiple distinct clusters, and the set of "variable" reactions). Sensitivity analysis shows the results are stable: total cluster counts scale with identity, but the reactome remains functionally closed, the ~100 genetically heterogeneous reactions persist within small fluctuations, and the mean functional clusters per reaction changes little from 65–90% (≈13–15) and increases at 95% (≈19.6) without altering the leading outliers.*

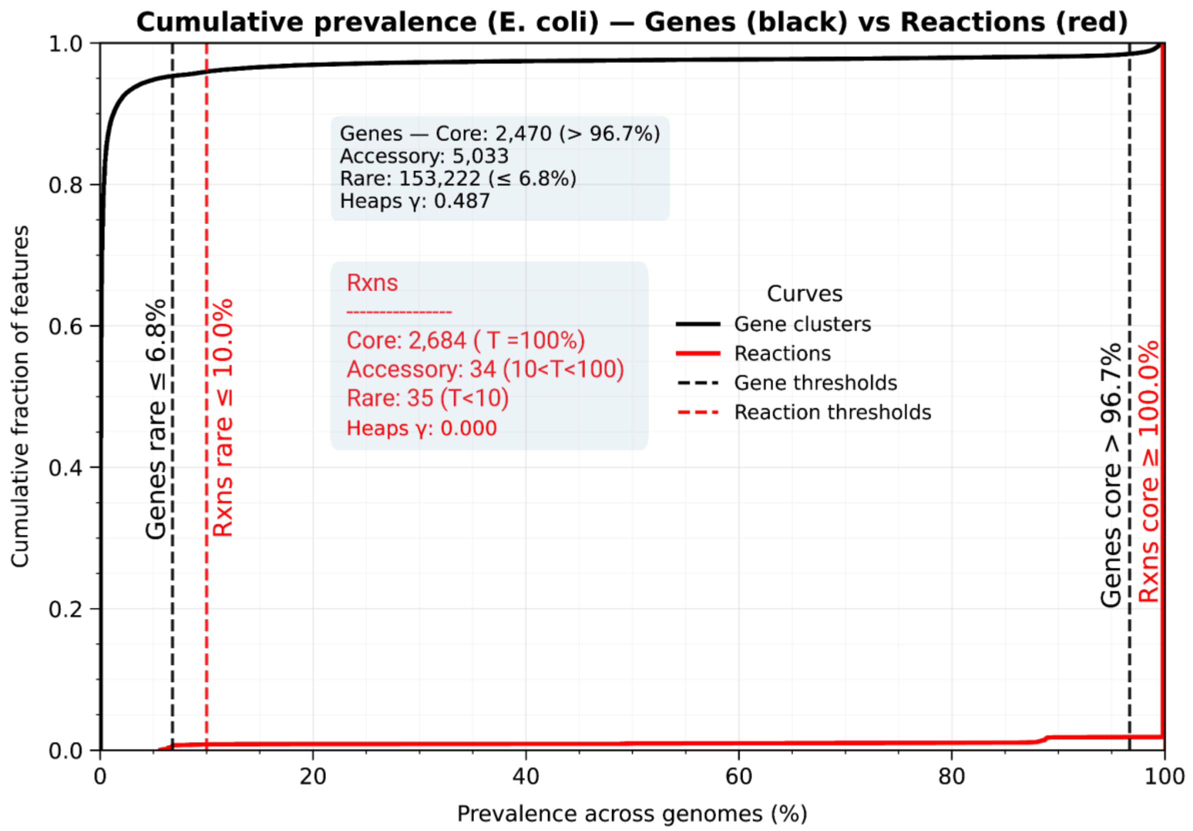

Fig. S2. Overlaid cumulative prevalence of gene clusters and reactions across E. coli genomes. *Black shows the cumulative distribution of gene-cluster prevalence; red shows the distribution of reaction prevalence derived from GEMs. Vertical dashed lines mark thresholds: genes—core >96.7%, rare <6.8%; reactions—core ≥100%, rare ≤10%; accessory is the remainder in each case. Inset boxes report the counts of core, accessory, and rare features and the pangenome/reactome openness (Heaps' γ).*

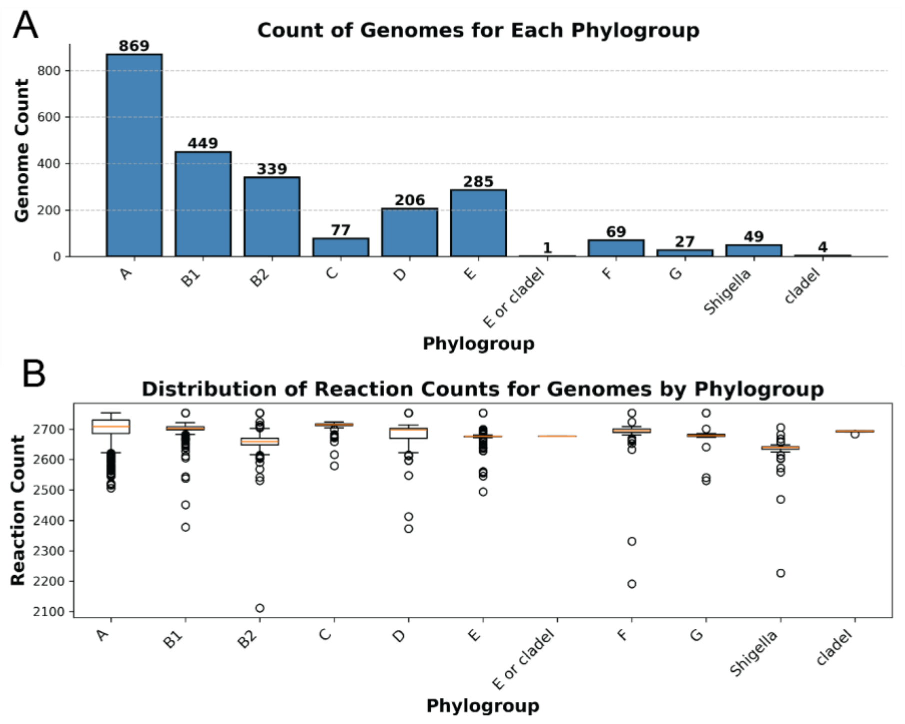

Fig. S3. Basic GEM information. (A) *A bar chart showing the distribution of genomes for which GEMs were reconstructed across different phylogroups.* ***(B)*** *A boxplot illustrating the average and range of reaction counts for GEMs within each phylogroup.*

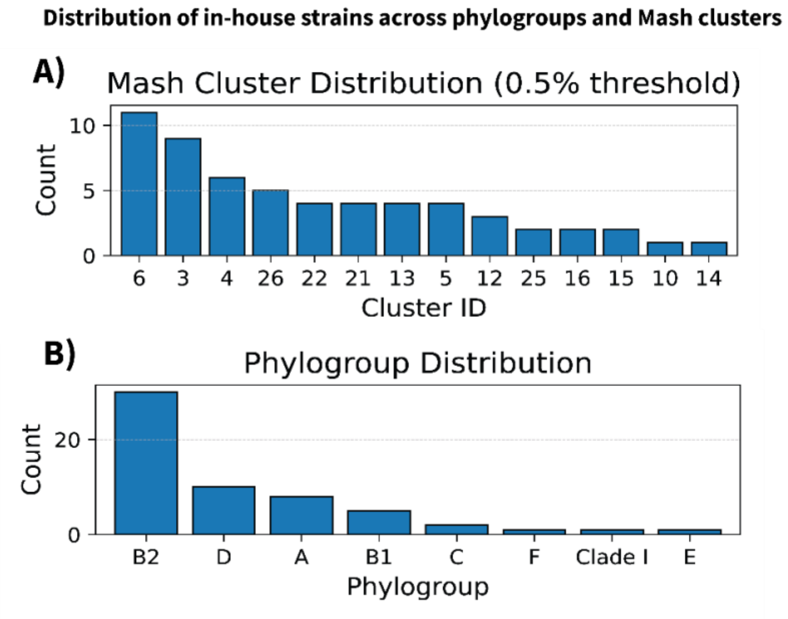

Fig. S4. Phylogenetic diversity of strains used for validation. (A) *represents distribution of strains across mash clusters drive from mash distance with a 0.5% cutoff. Cluster IDs are presented on X and the count of strains on Y axis. (****B)*** *represents distribution of strains across phylogroups. Phylogroups are presented on X and the count of strains on Y axis*

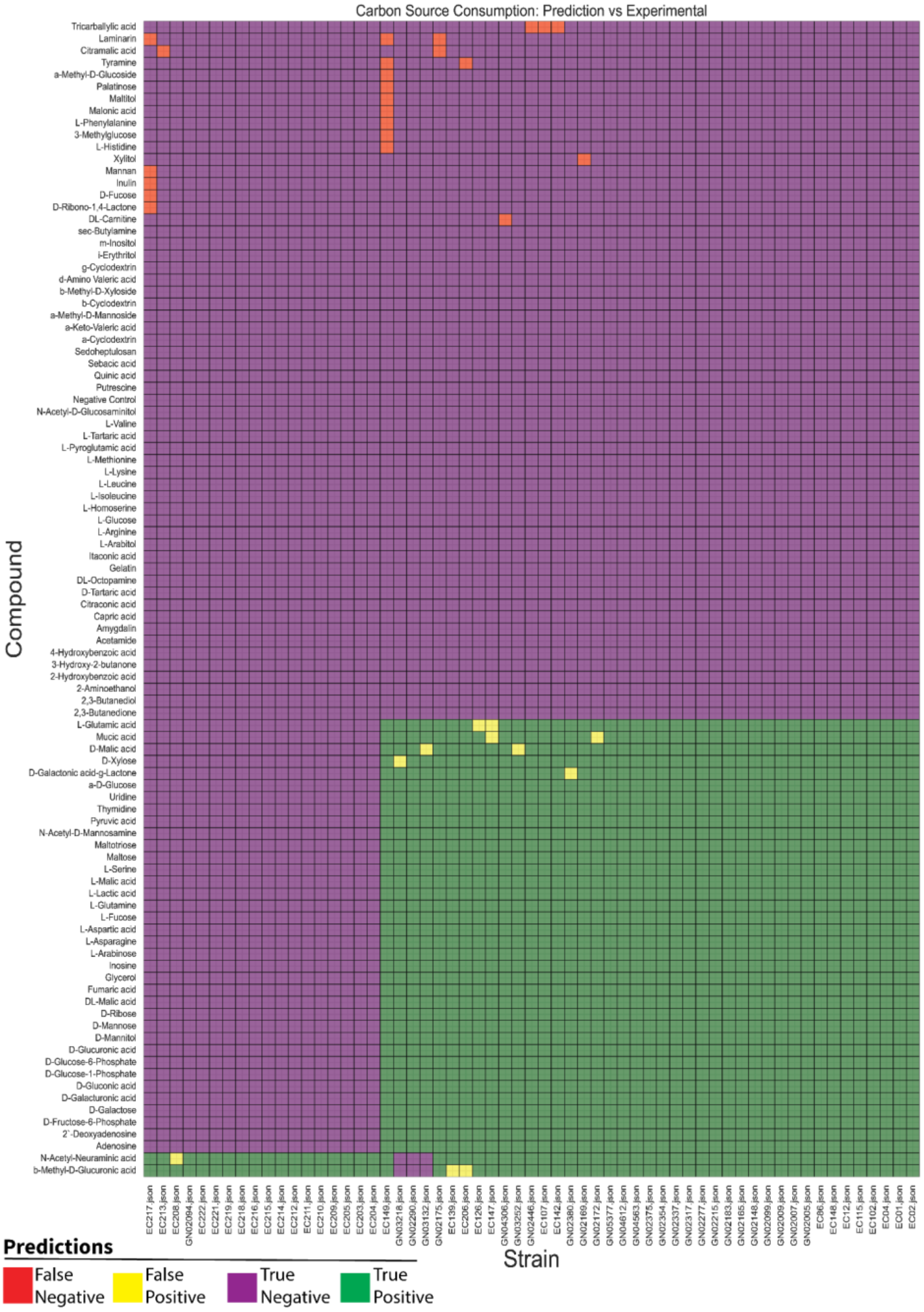

Fig. S5. GEM validation. *This Fig. is an enlarged version of Fig. 2A to improve readability. The growth of 59 strains was experimentally measured on 96 carbon sources, and the results were compared to the model's predictions for the same set of carbon sources. Green cells represent true positive predictions, where the model correctly predicts growth that matches the experimental phenotype. Purple cells represent true negative predictions, where the model correctly predicts no growth, consistent with the experimental results. Yellow cells represent false positives, where the model predicts growth, but the strain does not grow experimentally. Red cells represent false negatives, where the model predicts no growth, but the strain grows experimentally.*

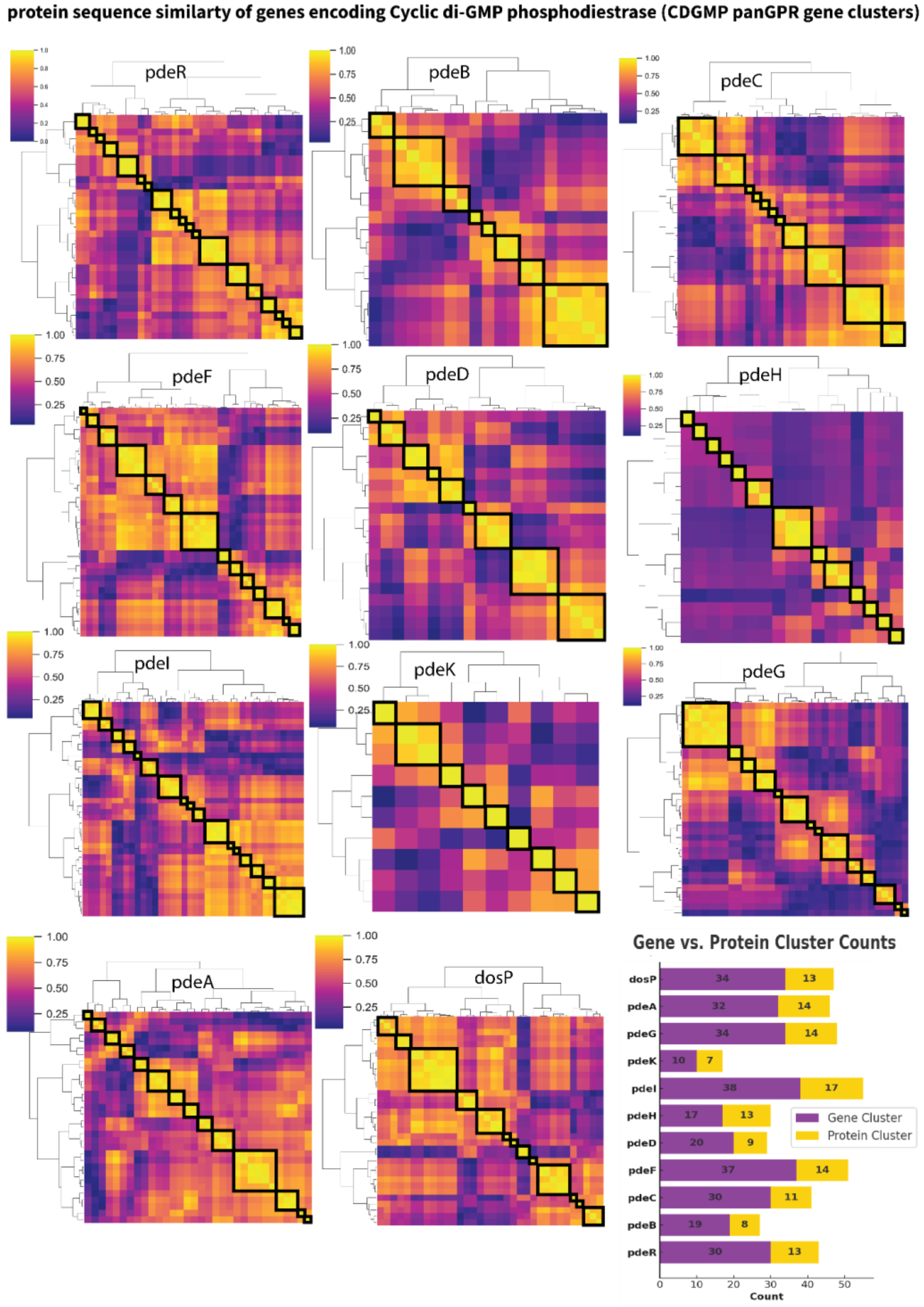

Fig. S6. Cyclic di-GMP phosphodiesterase protein sequence similarity. *Heatmap represents sequence similarity for Cyclic di-GMP phosphodiesterase genes, for each gene the longest variant was selected to perform a bidirectional BLAST for all pairs of genes, cells are color coded based on their similarity. The bar chart indicates the number of gene clusters with 80% similarity threshold for nucleotide sequences (purple) and corresponding translated amino acid sequences (yellow).*

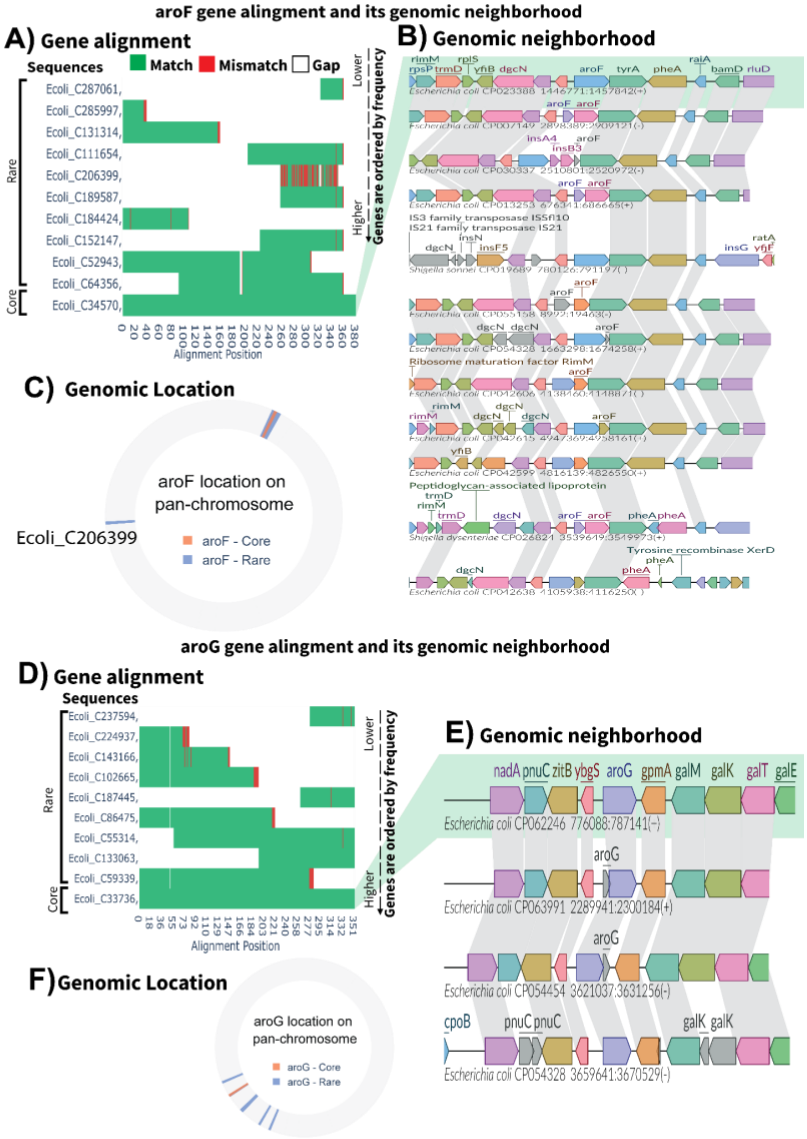

Fig. S7. Multiple sequence alignment and gene neighborhood for core, and rare genes of aroG and aroF isozymes. (A) *MSA of rare aroF genes to core aroH gene, genes are ordered based on frequency from bottom to top.* ***(B)*** *genomic neighborhood of core and rare aroF genes, homologous genes are highlighted across genomes.* ***(C)*** *Gene locations on pan-chromosome. Core genes are highlighted in orange and Rare genes are in blue. Genomes are aligned by the genes shared by all the genomes and only appear once.* ***(D)*** *MSA of rare aroG genes to core aroG gene, genes are ordered based on frequency from bottom to top. E) genomic neighborhood of core and rare aroG genes, homologous genes are highlighted across genomes. F) core genes are highlighted in orange and Rare genes are in blue. Genomes are aligned by the genes shared by all the genomes and only appear once.*

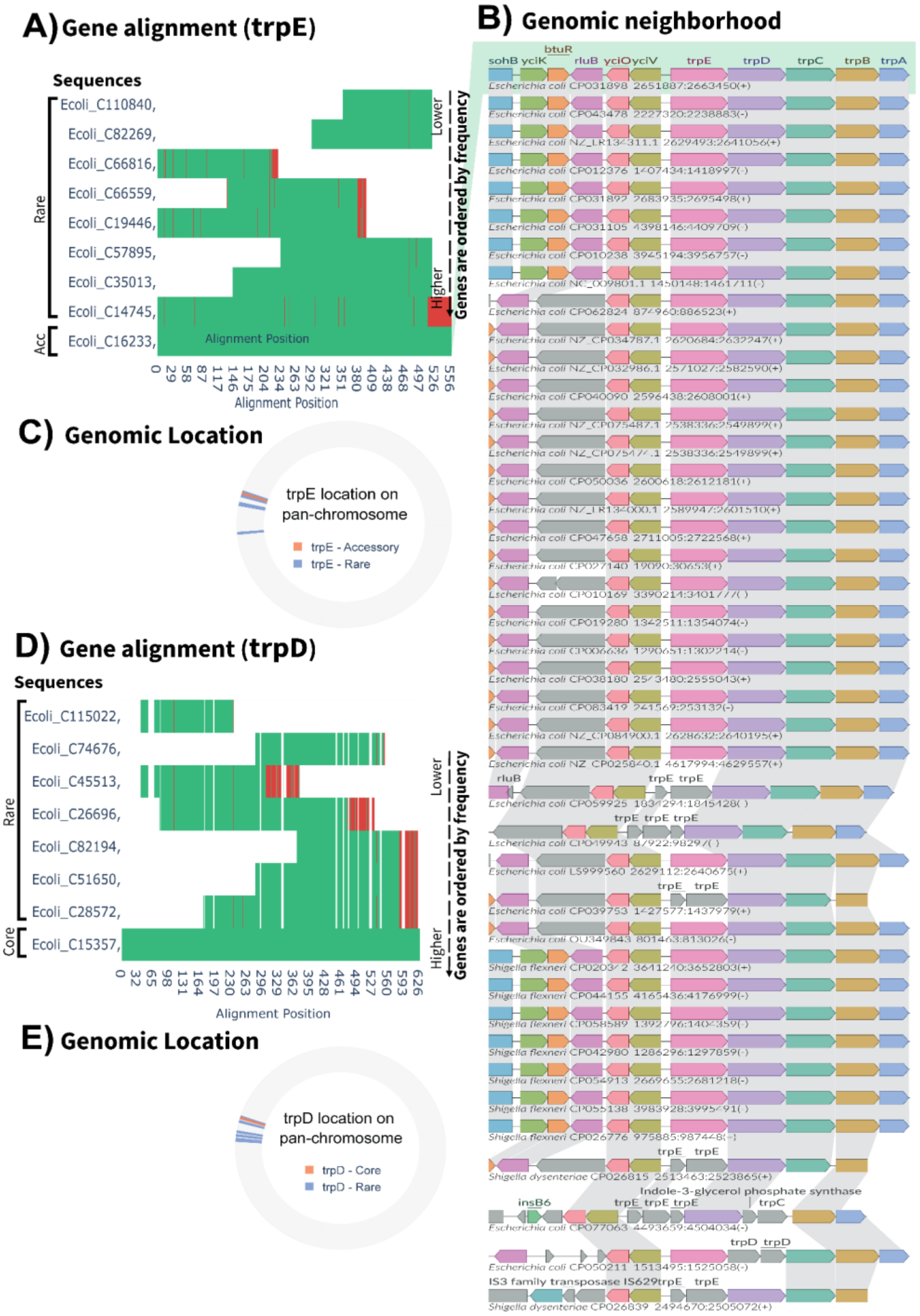

Fig. S8. Multiple sequence alignment and gene neighborhood for core, accessory, and rare genes of trpD and trpE isozymes. (A) *MSA of rare trpE genes to Accessory trpE gene, genes are ordered based on frequency from bottom to top.* ***(B)*** *genomic neighborhood of accessory and rare trpE genes and core and rare trpD genes, homologous genes are highlighted across genomes.* ***(C)*** *Gene locations on pan-chromosome. Accessory genes are highlighted in orange and Rare genes are in blue. Genomes are aligned by the genes shared by all the genomes and only appear once.* ***(D)*** *MSA of rare trpD genes to core trpD gene, genes are ordered based on frequency from bottom to top.* ***(E)*** *core genes are highlighted in orange and Rare genes are in blue. Genomes are aligned by the genes shared by all the genomes and only appear once.*

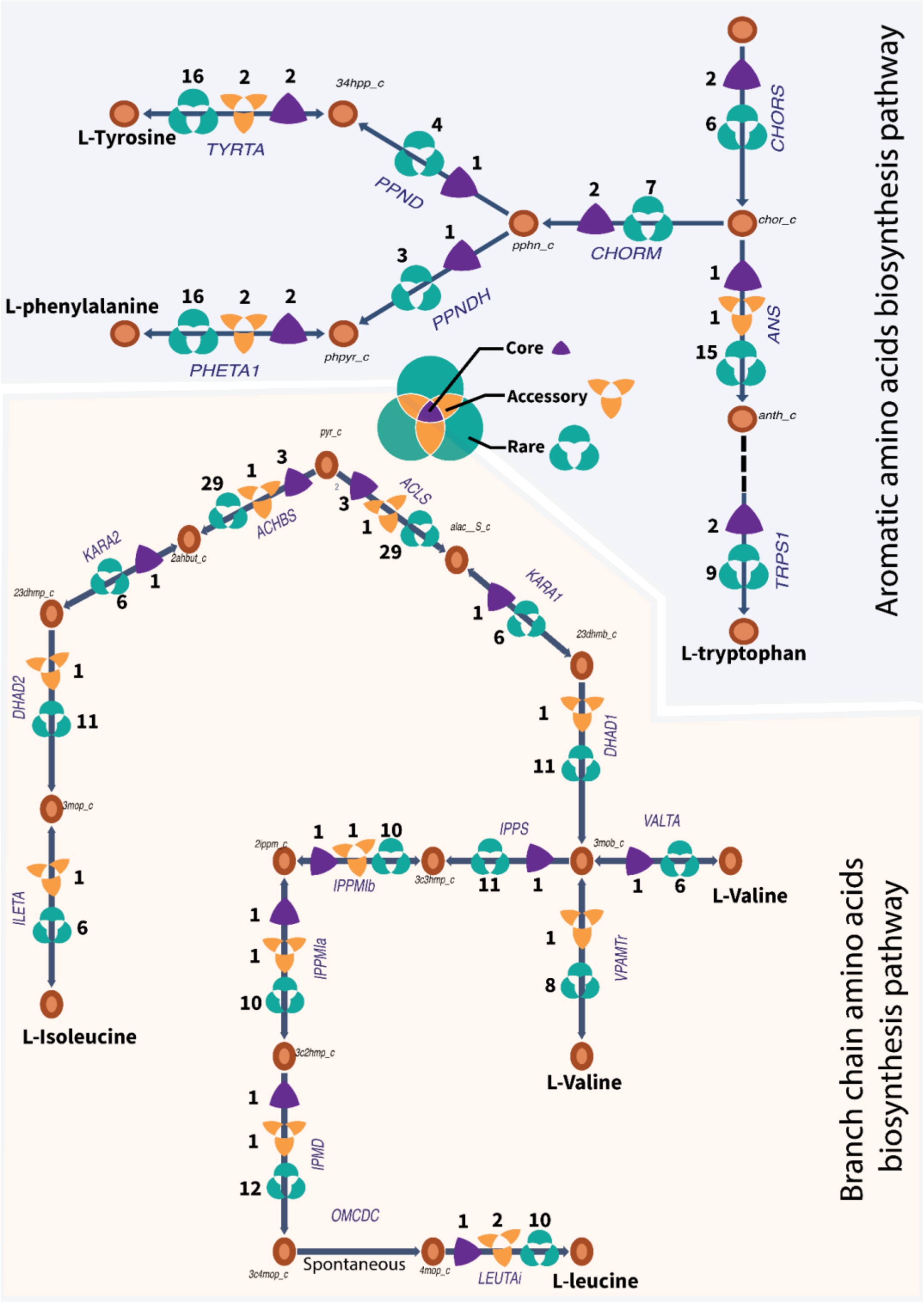

Fig. S9. Diversity of genetic basis of reactions in AAA and BCA biosynthesis pathways. *Metabolic pathways of AAA biosynthesis and BCA biosynthesis are annotated with the number of core, accessory, and rare genes coding each reaction across the pangenome.*

Supplementary Data

Data S1. *Complete genomes list and related metadata used in this study. The table lists the 2,377 complete bacterial genomes selected as targets for metabolic reconstruction, together with their associated metadata.*

Data S2. *Complete list of genome-scale metabolic models used as templates for generating the GPRs. The table lists the 70 published, manually curated genome-scale metabolic models used as reference templates for transferring gene–protein–reaction (GPR) associations to the target genomes. For each template, the following information is provided: model identifier (Model), source organism and strain (Organism), and model size in terms of number of metabolites (Metabolites), reactions (Reactions), and genes (Genes). The collection comprises 58 Escherichia coli models (including the core model e_coli_core and the most recent reconstructions iJO1366 and iML1515), 8 Shigella spp. models, 2 Salmonella models (including the pan-reactome iYS1720), 1 Klebsiella pneumoniae model (iYL1228), and 1 Yersinia pestis model (iPC815).*

Data S3. *List of formulated reactions from the KEGG database. The table reports, for each target genome, the set of genes whose orthologs are missing from the BiGG-based template reconstructions and for which a reaction was therefore formulated de novo from KEGG annotations (153,733 model-gene entries covering 2,154 target genomes, 272 unique genes, and 211 unique EC numbers / KEGG reactions).*
